# A Comprehensive Feature Analysis of the Fetal Heart Rate Signal for the Intelligent Assessment of Fetal State

**DOI:** 10.3390/jcm7080223

**Published:** 2018-08-20

**Authors:** Zhidong Zhao, Yang Zhang, Yanjun Deng

**Affiliations:** 1 Hangdian Smart City Research Center of Zhejiang Province, Hangzhou Dianzi University, 310018 Hangzhou, China; mynameiszhangyang@gmail.com (Y.Z.); dengyanjun79@gmail.com (Y.D.); 2 College of Electronics and Information, Hangzhou Dianzi University, 310018 Hangzhou, China; 3 School of Communication Engineering, Hangzhou Dianzi University, 310018 Hangzhou, China

**Keywords:** fetal state assessment, fetal heart rate, feature analysis, classification

## Abstract

Continuous monitoring of the fetal heart rate (FHR) signal has been widely used to allow obstetricians to obtain detailed physiological information about newborns. However, visual interpretation of FHR traces causes inter-observer and intra-observer variability. Therefore, this study proposed a novel computerized analysis software of the FHR signal (CAS-FHR), aimed at providing medical decision support. First, to the best of our knowledge, the software extracted the most comprehensive features (47) from different domains, including morphological, time, and frequency and nonlinear domains. Then, for the intelligent assessment of fetal state, three representative machine learning algorithms (decision tree (DT), support vector machine (SVM), and adaptive boosting (AdaBoost)) were chosen to execute the classification stage. To improve the performance, feature selection/dimensionality reduction methods (statistical test (ST), area under the curve (AUC), and principal component analysis (PCA)) were designed to determine informative features. Finally, the experimental results showed that AdaBoost had stronger classification ability, and the performance of the selected feature set using ST was better than that of the original dataset with accuracies of 92% and 89%, sensitivities of 92% and 89%, specificities of 90% and 88%, and F-measures of 95% and 92%, respectively. In summary, the results proved the effectiveness of our proposed approach involving the comprehensive analysis of the FHR signal for the intelligent prediction of fetal asphyxia accurately in clinical practice.

## 1. Introduction

Delivery is one of the most important events in women's lives and having a healthy baby is a common desire. When the fetus suffers a pathological condition, timely intervention can prevent permanent damage and improve the birth quality of the population. However, the fetal state is influenced to varying degrees by several risk factors related to the pregnant woman and the external environment, and the rate of neonatal morbidity and mortality is increased in case of prolonged and severe reduction in oxygen supply [1]. Therefore, an effective technique used to monitor the fetal situation when necessary is needed.

In clinical practice, cardiotocography (CTG), a technique for recording the fetal heart rate (FHR) signal and uterine contraction (UC) activity, is currently the most routinely used method for antepartum and intrapartum monitoring of fetal well-being [1]. CTG, also known as electronic fetal monitoring (EFM), can assist obstetricians in identifying fetal hypoxia and thereby prevent several abnormal outcomes, such as metabolic acidosis, congenital heart defect, and even death [2]. Unfortunately, FHR is interpreted visually by clinicians via the naked eye, which causes not only intra-observer (one expert at different times), but also inter-observer (different experts at the same time) variability. In addition, the high false positive rate of the FHR signal is considered the main reason behind the increasing rate of caesarean sections (CSs), and unnecessary operative deliveries. To overcome the above drawbacks, researchers have attempted to design computer-aided analysis systems for assisting clinicians in diagnosing the fetal state, through extracting useful information from the FHR signals [3].

Different research institutes have proposed relevant guidelines that are limited and insufficient, e.g., the International Federation of Gynecology and Obstetrics (FIGO) [4] and the National Institute of Child Health and Human Development (NICHD) [5], etc. These guidelines barely consider (and the interrelated systems calculate) basic morphological features of the FHR signal, namely, the baseline estimation, detection of acceleration and deceleration pattern, and variability in short and long term. Even though the guidelines are widely used in the delivery ward, the FHR signal is interpreted poorly and differences in fetal state assessment among obstetricians still exist [6]. 

In recent decades, related studies have proved the effectiveness of analyzing features originated from different aspects in the diagnosis of fetal distress. Krupa et al. introduced the detail coefficients using empirical mode decomposition (EMD) and employed the support vector machine (SVM) to classify the FHR signals with accuracy (Acc) of 87% [7]. Spilka et al. investigated the influence of nonlinear features (complexity, entropy, and fractal dimension) on classification and obtained sensitivity (Se) and specificity (Sp) of 73% and 76%, respectively [8]. Doret et al. applied a univariate analysis method for the frequency domain and nonlinear parameters [9]; the former had the higher area under the curve (AUC) value of 0.81 ± 0.07 with a 95% confidence interval (CI). Comert et al. proposed a prognostic model, based on image-based time-frequency (IBTF) features and least square SVM (LS-SVM), which achieved better performance than conventional feature-based methods [10]. 

The antenatal fetal non-stress testing (NST) and intrapartum EFM surveillance have been widely applied in the diagnosis of fetal state by obstetricians in clinical practice. Both the approaches utilized FHR signals, which performed well during the antepartum and intrapartum stages, respectively. Low et al. studied 290 mature pregnancies and proved that there were relationships between the antepartum and intrapartum FHR signals [11]. For example, there was a significant relationship in the baseline and its variability, while there was no correlation in accelerations and decelerations. Furthermore, Low et al. demonstrated that the analysis method used in one of them could be equally used in another stage due to their relationships. Although many of the previous methods as described in References [7,8,9] treated the antepartum signal as an analysis object, several researchers illustrated that these features could be extensively employed in the intrapartum stage citing a freely available database consisting of intrapartum FHR recordings since 2014 [12], which were also employed in this work.

Furthermore, inspired by the rapid development of the adult heart rate variability (HRV) field, the fetal HRV signal has also proved to perform well in identifying fetuses at risk of diseases (e.g., sudden infant death syndrome (SIDS)), by providing detailed physiological information about the fetus [13]. Recent studies involving HRV signals (obtained from electrocardiogram (ECG) directly or CTG indirectly), have revealed that HRV can reflect fetal hypoxia and acidosis, pathological conditions that are closely associated with the increased risk of neonatal morbidity and mortality [14]. 

**Figure 1:**
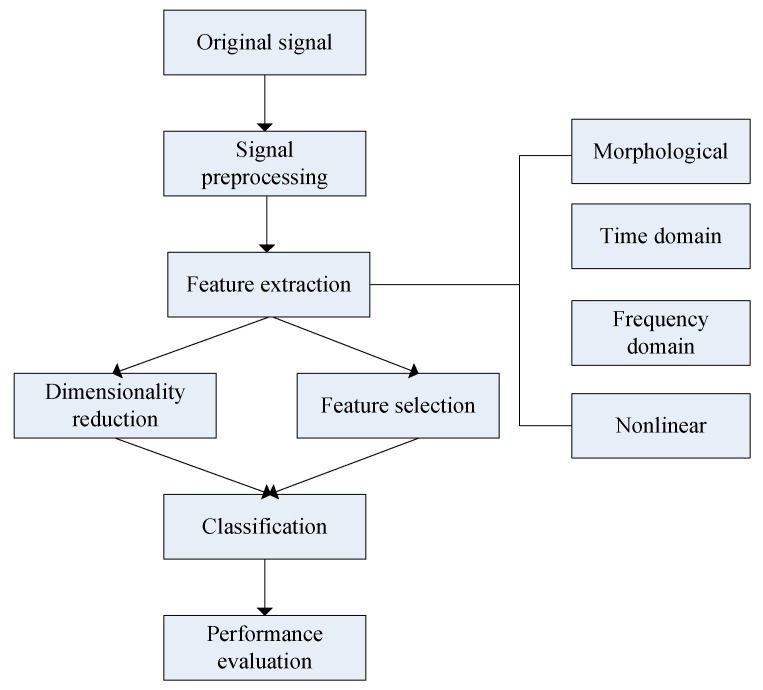
A schematic presentation of our proposed approach.

In this work, we proposed a novel computerized analysis method using FHR signals, which aims to provide medical decision support based on advanced signal process methods and machine learning (ML) algorithms. The procedure is illustrated in Figure 1 and consists of the following major steps: Signal preprocessing, feature extraction and selection (dimensionality reduction), classification, and performance evaluation. The work has several innovative aspects: (i) We took the FHR signal into consideration comprehensively and extracted a great number of relevant features, including morphological, time and frequency, and nonlinear domains; (ii) feature selection algorithms were applied to determine which features are more informative for classifying the fetal pattern; and (iii) ensemble learning was used to discriminate between two fetal states: Normal and pathological.

The rest of the paper is organized as follows: Section 2 presents the database and a detailed description of the overall methodology for FHR signal, especially feature analysis. Section 3 gives the corresponding results of the different steps and Section 4 discusses the classification results of the dataset. Section 5 concludes the work and proposes the direction of future work.

## 2. Materials and Methods

### 2.1. Data Description

The data used in the study were obtained from CTU-UHB, an open-access intrapartum CTG database comprising a subset of 9164 intrapartum CTG recordings that were acquired between the years 2009 and 2012 in the obstetrics ward of the University Hospital in Brno, Czech Republic [12,15]. Chudáček et al. elaborately selected the final 552 CTG signals to constitute this database with clinical as well as technical considerations. Three sets of 102, 412, and 35 records were acquired by means of scale electrode (ECG), ultrasound pressure (CTG), and both, respectively. The last three records of this database contained unavailable information. The main types of information and their respective distributions are depicted in Table 1 and interested readers can refer to a more detailed characterization in Reference [12]. All records were sampled at 4 Hz using a recording device. In this work, we chose a signal length of 20 min (4800 samples) for continuous processing.

Two common types of annotation are widely accepted according to References [16,17]: (i) The pH or BE (base deficit) of neonatal umbilical artery blood measured immediately after the delivery can be viewed as an objective annotation; (ii) both expert evaluation of the fetal pattern and measurement of the newborn (e.g., Apgar score in 1st and 5th min) in the delivery ward are subjective annotations. In this work, pH was selected as the gold standard to assign the fetal state into one of two classes to reduce subjective error. A pH below 7.15 was agreed as pathological and a pH greater than or equal to 7.15 was classified as normal; thus, the database contained 447 normal and 105 abnormal (pathological) FHR recordings. 

### 2.2. Software Interface

**Figure 2:**
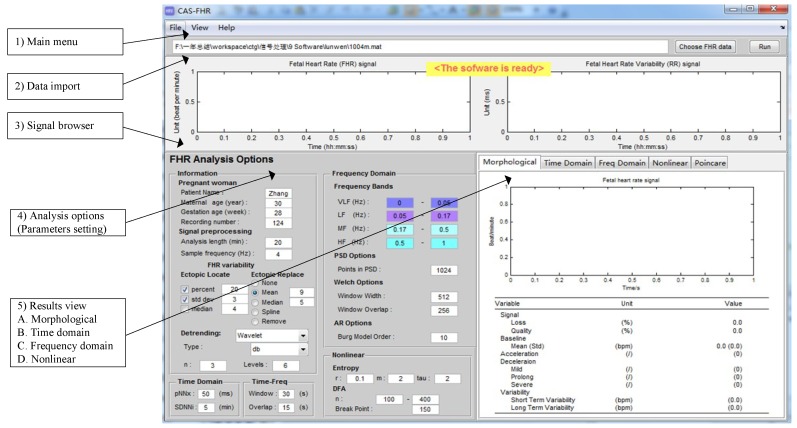
The graphical user interface of the computerized analysis software of the FHR signal (CAS-FHR) is divided into five segments: (**1**) Main menu; (**2**) Data import; (**3**) Signal browser; (**4**) Analysis options (Parameter settings); and (**5**) Results viewer. The results viewer can be further divided into morphological, time and frequency domain and nonlinear results.

In this work, the user-friendly CAS-FHR (computerized analysis software of the FHR signal) software interface was developed by means of the MATLAB graphical user interface development environment (GUIDE) and allowed the researchers to interact with the software through several edit boxes and buttons. The built-in-functions (BIFs) were independent and involved different analysis stages, including data import, signal preprocessing and feature extraction (morphological, time and frequency domain and nonlinear features). Moreover, the users could arbitrarily set and adjust input parameters as required. Then, the result was displayed on the interface in various forms (i.e., as a digital table and a figure) and could be saved for further study along with the information regarding the pregnant woman (MA, GA, etc.). Figure 2 shows the software interface with the advanced settings.

### 2.3. Signal Preprocessing

In clinical practice, during the recording process using Doppler ultrasound, the FHR signal contains many artifacts or spikes due to maternal and fetal movements or transducer displacement [1]. Therefore, before further analysis, we eliminated noise to obtain a relatively pure signal for more accurate results, as described in Reference [18]. In this work, we employed a preprocessing algorithm involving three steps. Assume *x*(*i*) is an FHR signal with unit of beats per min (bpm) and a sample frequency of 4 Hz, where *i* = 1,2, ..., *N* and *N* is the number of samples.

1. A stable segment is chosen as the starting point; in such a segment, five adjacent samples do not differ by more than 10 bpm, and missing data are excluded when the length of *x*(*i*) = 0 is equal to or more than 10 s.
2. Values of *x*(*i*) ≤ 50 or *x*(*i*) ≥ 200 are considered data spikes and are removed using linear interpolation.
3. We interpolate *x*(*i*) using spline interpolation again when the difference of *x*(*i*) and *x*(*i* − 1) exceeds 25 bpm, a value used to define an unstable segment.

**Figure 3:**
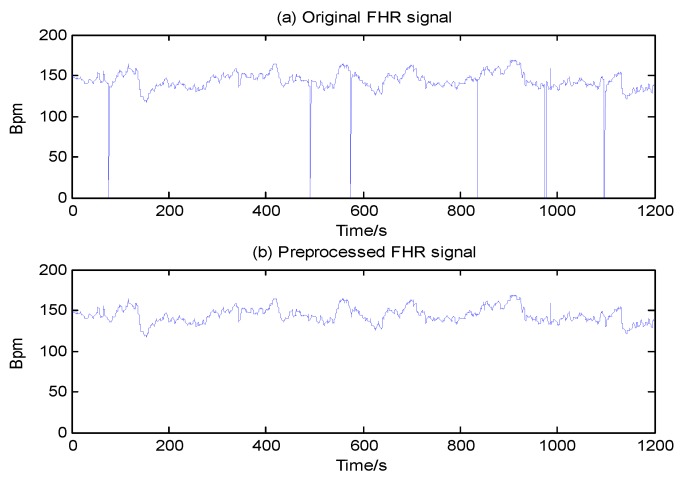
Signal preprocessing of No. 1001 fetal heart rate (FHR) recording (internal database number). (**a**) Original FHR signal; (**b**) Denoised FHR signal.

Twenty minutes (*N* = 4800 samples) of signal length was the target used for further continuous processing in this paper. Taking the signal labeled No. 1001 as a typical example, the result of this artifact removal scheme is presented in Figure 3.

### 2.4. Feature Extraction

As shown in Figure 1, feature extraction after signal preprocessing was the most important step in analyzing FHR signal and assessing fetal state. To the best of our knowledge, the extracted features used in this work represented an almost complete collection of features that have been employed for the automatic evaluation of the FHR signal in previous studies.

#### 2.4.1. Morphological

According to the common FIGO guidelines motivated by the routine application of the FHR signal by obstetricians and midwives in recent decades, baseline, acceleration, deceleration and variability are basic morphological features [4]. Given they represent macroscopic properties of the FHR pattern and are easily visible to the naked eyes of experts, these four features are most frequently used in clinical settings. 

Baseline (BL) is generally considered the important feature used in FHR pattern recognition because the other morphological features rely on its value [19]. BL can also be considered the resting level of the FHR, i.e., the mean level of the signal when it is stable, and acceleration and deceleration are absent [4,20]. The BL is classified as reassuring, non-reassuring and abnormal when the mean of the BL is in the ranges of 110–160 bpm, 100–109 bpm or 161–180 bpm, and less than 100 bpm or more than 180 bpm, respectively [4,20]. Accelerations (ACCs) are temporary increases in the FHR above the BL by 15 bpm or more, for 15 s or longer. Decelerations (DECs) are temporary decreases in FHR below the BL by more than 15 bpm, for 15 s or longer. DECs can be classified into three types based on duration time: Mild, prolonged, and severe are defined as less than 120 s, between 120 and 300 s, and more than 300 s, respectively [4,20]. 

According to the definition described above, the software implemented one-dimensional filtering and applied a sliding window to the FHR sequence. This filtering replaced the center value in the window with the average value of all the points within the window.

Therefore, the first set of extracted features was as follows:

Set_1: {meanBL, sdBL, minBL, maxBL, ACC, DEC_mild, DEC_prolong, DEC_severe}.

As mentioned above, the basic features of the FIGO guidelines are considered important and necessary for the development of computerized systems for the automatic prediction of the fetal state [21]. In addition, other features originated from the adult HRV signal of different domains have proven to be equally useful. The mutual relationship between the sympathetic nervous system (SNS) and the parasympathetic nervous system (PSNS) of the fetus can be reflected in clear variations in the fetal HRV signal. More specifically, regarding the pathological state of the fetus, stimulation of the SNS results in a decrease in heart rate (HR), while stimulation of the PSNS results in an increase in HR [22]. During the periods of stress, such as the prolonged DEC or at the time of UC, the fetal heart pumping activity would be improved due to the SNS serving as a compensatory mechanism, as reflected in the FHR signal variations. Unlike an adult HRV using the ECG signal, the FHR obtained from CTG recording has no real RR (beat-to-beat) interval [23]. Thus, before extracting linear and nonlinear features derived from the adult HRV parameters, we firstly needed to change the FHR signal to epoch-to-epoch variation (i.e., fetal HRV signal), with a unit of millisecond like others (e.g., Reference [24]), expressed as Equation (1).

(1) $$\mathrm{RR}=\frac{60,000}{\mathrm{FHR}}\;$$

#### 2.4.2. Time Domain

When the fetus does not have a pathological heart condition, all beats can be considered normal, and the distance between two normal beats is described as NN. Inspired by commonly used parameters in the field of adult HRV, we computed several statistical measures of the fetal HRV signal in the time domain [13]. The maximum, minimum, mean and median values of the RR interval were four basic attributes. Other parameters include the standard deviation of the NN (SDNN), calculated on the chosen HRV segment, which reflects all the cyclic components responsible for variability in the period of recording and has two variants (SDANN and SDNNi with the same fractional segments); the root of the mean squared differences (RMSSDs) of consecutive RR intervals; NNx, which computes the number of successive NN pairs that differ by more than *x* ms; pNNx, which gives the percentage of NNx to the total number of beats; Tri, which represents the HRV triangular index and calculates the samples in a bin and the location of the bins using histogram analysis; and TINN, the triangular interpolation of the NN interval histogram. Many researches have recently been proposed to prove the effectiveness of such parameters and have been provided as a clinical basis. For example, Torres et al. designed a case-control study to analyze the HRV signal at rest and during aerobic exercise in healthy people and cardiac patients using time domain parameters [25]. The experimental result demonstrated that the healthy group showed a significant decrease in SDNN and pNN50. Other features reflect slight changes in fetal behavior that are difficult to observe using the naked eye, including short-term variability (STV), interval index (II), long-term irregularity (LTI), delta value and total delta value. The corresponding calculation formula can be found in reference [26]. A total of 17 linear time domain attributes were extracted from the HRV signal and used for classification.

Therefore, the second set of extracted features in the time domain of HRV was as follows:

Set_2: {meanRR, minRR, maxRR, medianRR, SDNN, SDANN, SDNNi, RMSSD, NNx, pNNx, STV, II, LTI, delta, delta_total, Tri, TINN}.

#### 2.4.3. Frequency Domain

For adult HRV, several spectral methods have been proposed [27]. However, there is no generally acknowledged use of frequency bands in the analysis of the FHR signal. In this work, we adopted suggestions from Reference [28] for use in frequency partitioning. The frequency range was divided into 4 bands, and the power spectral of the signal in each one of these was computed as follows: Very low frequency (VLF, 0–0.03 Hz) band, which is related to very low control mechanisms and presents nonlinear characteristics; low frequency (LF, 0.03–0.15 Hz) band, which is mainly associated physiologically with neural sympathetic fetal activity; movement frequency (MF, 0.15–0.50 Hz) band, which is correlated with physical activity (e.g., fetal movements and maternal breathing); and high frequency (HF, 0.50–1.00 Hz) band, which reflects fetal breathing. Motivated by the ratio of LF/HF in the field of adult HR, the ratio of energies in the bands was also computed as: Ratio_Band = LF/(MF + HF), which is believed to quantify the balance of activity between the two-autonomic nervous system (ANS) branches (SNS and PSNS) [29]. In this work, the software provided three ways to calculate the power spectral density (PSD), including Fast Fourier Transform (Welch), the auto-regressive (Burger) model, and Lomb Scargle (LS). The analysis parameters of frequency domain could be set arbitrarily in the software interface. 

Therefore, the third set of extracted features in the frequency domain of HRV was as follows, where Power_VLF represented the energy of the VLF band using PSD, Percent_VLF represented the percentage of VLF in the total energy band, and the other three features had the same meanings:

Set_3: {Power_VLF, Power_LF, Power_MF, Power_HF, Power_Total, Percent_VLF, Percent_LF, Percent_MF, Percent_HF, Ratio_Band}.

#### 2.4.4. Nonlinear

The nonlinear parameters extracted from the fetal HRV signal were chosen based on adult HRV studies [8,30].

The fractal dimension (FD) is one of the useful estimators of HRV kinetics, and several techniques for estimating the waveform FD have been proposed [31,32]. The Higuchi method was chosen in this work and calculates the FD from the chosen length of the HRV signal [33]. Assuming an original signal *x*(*1*)*, x*(*2*), ..., *x*(*N*) of length *N*, a new signal*$X_{k}^{m}$* is constructed as follows: *X*(*m*)*, X*(*m* + *k*), *X*(*m* + *2k*), ..., *X*(*m* + [(*N* − *m*)/*k*]) (*m* = 1,2, ..., *k*), where [] denotes the Gauss’ notation, *m* defines the initial time, and *k* is the time interval. *k* represents the time displacement, and the number of new created subsets is equal to *k*. Then, for each *m*, the length *L_m_*(*k*) of $X_{k}^{m}$ is computed. The length of the curve for time interval *k* is <*L*(*k*)>, which is defined as the average value over *k* sets of *L_m_*(*k*). The computed curve length <*L*(*k*)> for a different value of *k* is related to the FD *D* by the exponential formula <*L*(*k*)>∝*k^−D^*. The FD (noted as FD_Hig) is estimated as the slope of a fitted regression curve to the log-log plot of <*L*(*k*)> versus *k* [10].

(2) $$<L(k)>=\sum_{m=1}^{k}\frac{L_{m}(k)}{k}\;$$

The entropy index denotes the behavior of a nonlinear signal and quantifies the underlying randomness [34]. The approximate entropy (ApEn) and the sample entropy (SampEn) are most frequently used and are effective in deciding the fetal state [35,36]. The former approximately equals the average of the natural logarithm of the conditional probabilities that sequences of length *m* are close to each other, within a tolerance *r*. The latter is a slightly modified version of ApEn and overcomes the inherent disadvantages. This is mainly used because conditional probabilities are not estimated by a template approach and self-matches are excluded. This procedure requires that only one template finds a match of length *m* + 1. In this work, the parameters used for ApEn and SampEn estimation were the embedding dimension *m* = 2 and the tolerance *r* = 0.15 × SD (the standard deviation of the RR time series). The values of *m* and *r* could be set arbitrarily in the software interface. A discrete signal *x_n_* of length *N* is separated into a subset of *m* length vectors *u_m_*(*i*), and the numbers of vectors *u_m_*(*i*) and *u_m_*(*j*), which are close to each other in a Euclidean sense *d*[*u_m_*(*i*), *u_m_*(*j*)] ≤ *r*, are conveyed by the number $n_{i}^{m}$(*r*). The following equations are shown as (3) to (6). 

(3) $$\mathrm{ApEn}(m,r,N)=\underset{N\to \infty }{\lim}{[\Phi }^{m}(r)-\Phi^{m+1}(r)]\;$$

(4) $$\Phi^{m}(r)=\frac{1}{N-m+1}\sum_{i=1}^{N-m+1}\ln C_{i}^{m}(r)\;$$

(5) $$C_{i}^{m}(r)=\frac{n_{i}^{n}}{N-m+1}\;$$

(6) $$\mathrm{SampEn}(m,r,N)=\underset{N\to \infty }{\lim}-\ln\frac{C^{m+1}(r)}{C^{m}(r)}\;$$

One of the important nonlinear parameters is the Lempel Ziv Complexity (LZC) [37,38]. This method examines recurring patterns that are obtained in the continuous signal irrespective of time. A random signal has rarely repeated individual patterns; thus, the signal complexity is high. The opposite is true for the periodic signal. A time series, *x*(1), *x*(2),...,*x*(*n*), is first encoded to constitute a sequence *S* such that the values of signals are equal; *x*(*i* + 1) *= x*(*i*) is encoded by 2 and increases, *x*(*i* + 1) > *x*(*i*) is encoded by 1 and decreases, *x(i* + 1) < *x*(*i*) is encoded by 0, where the quantification level *p = 0*. The second step is computing the distinct patterns in *S*; the complexity *c*(*n*) is increased by 1 for each new pattern. When the last element of *S* is reached, *c*(*n*) is still increased by 1; obviously *c*(*n*) depends on the number of data points *n*. Finally, the normalization form is designed to avoid the dependence on the length of the original sequence. The normalized *C*(*n*) is described by Equation (7). The primary purpose of ternary encoding is to avoid a dependence of the results on the normalization procedures and quantification criteria. 

(7) $$C(n)=\frac{c(n)\times \log_{2}(n)}{n}\;$$

In addition, the Hurst parameter for intrapartum fetal HRV analysis [9]; the short/long scale exponent (alpha) of the detrended fluctuation analysis (DFA) [39]; and the average acceleration capacity (AAC), the acceleration phase-rectified slope (APRS), the average deceleration capacity (ADC) and the deceleration phase-rectified slope (DPRS) as obtained from the phase-rectified signal average (PRSA) are also extracted from the HRV signal [40,41]. Furthermore, a Poincare plot is used to represent the correlation of the signal itself and to aid clinicians in determining outliers and the overall quality of the signal [42]. Each RR interval is plotted as a function of the previous interval (the relationship between *x*(*i*) and *x*(*i* + 1)) and the standard deviation of two axes (SD1, SD2) is motivated by the geometric HRV representation. Due to space limitation, details were omitted but can be found in the cited publications. 

Therefore, the fourth set of extracted features in the nonlinear field of HRV was:

Set_4: {FD_Hig, ApEn, SampEn, LZC, Hurst, alpha, AAC, ADC, APRS, DPRS, SD1, SD2}.

After extracting the comprehensive features, we needed to do some preprocessing on the obtained dataset consisting of 47 features (from Set_1 to Set_4, marked as Set_Complete) before further analysis. For the fetal heart rate pattern (HRP) functional signal in the prenatal period, Hoyer et al. demonstrated that there existed a clear relationship between the gestational age (GA) and the extracted parameters from FHR signals [43]. We considered the fact that the parameters should be adjusted to eliminate the bias. Therefore, to obtain reliable results, we normalized all the parameters by performing linear regression based on GA, which is available in the open-access database (Table 1), inspired by the work of Magenes et al. [44]. Further, we also conducted the normalization process using the common minimum-maximum scaling method and compared their performance.

### 2.5. Feature Selection/Dimensionality Reduction and Classification

#### 2.5.1. Feature Selection/Dimensionality Reduction

The above feature extraction algorithms created a dataset combined with many features (47). Some of the features may have not been as informative as expected and have even conveyed overlapping information. In other words, not all the extracted features were necessary for classification or for aiding the obstetrician’s assessment of fetal state. Therefore, feature selection (or dimensionality reduction) is used to determine the features that contain more valuable information for the application of the classification problem [45].

From the clinician’s point of view, all features were submitted to the Mann-Whitney-Wilcoxon statistical test (ST), and we determined the significant differences according to the *p*-value [46]. 

The feature selection algorithm could then be used to decrease the training time for building a classifier and simultaneously retain the class discriminatory information. That is, the choice of a suitable subset of the features could increase computational efficiency and reach near-optimal performance regarding assessment of the fetal state. In this work, we ranked the features based on the value of the area under of the ROC (receiver operating characteristic) curve (AUC) and determined the best individual features [47]. Dimensionality reduction has the same effect, and principal component analysis (PCA) is generally used in biomedical applications [48]. 

#### 2.5.2. Classification and Performance Evaluation

In this work, we used the WEKA (Waikato Environment for Knowledge Analysis) data mining software to execute the final classification stage [49]. Many ML algorithms have been experimented to compare their performance, e.g., Naïve Bayes, k nearest neighbor, discriminate analysis, etc. However, due to the space restriction, and more importantly, the primary goal of the work was the comprehensive feature analysis method; we only selected three representative algorithms which possessed stronger classification capacity among the numerous tested ML algorithms, including C4.5 decision tree (DT) [50], SVM [51], and adaptive boosting (AdaBoost) [52]. 

Citing the low number of instances in the pathological class (105 of the total of 552), a ten-fold cross validation (CV) was applied to obtain more reliable results. The training set contained 497 (402 normal and 95 pathological) recordings, while the test set contained 55 (45 normal and 10 pathological) recordings.

Finally, the confusion matrix and some measurements (such as Acc, Se, and Sp) calculated from the elements were conventionally used in medical field [53], as shown in Table 2. Unfortunately, due to the existence of a high imbalance between the fetal classes for the used database (105 normal and 447 pathological cases), we chose other alternative indicators to evaluate the classification performance.

1. F-measure (FM, Harmonic mean):
  (8) $$\mathrm{FM}=\frac{2\cdot \mathrm{Precision}\cdot \mathrm{Recall}}{\mathrm{Precision}+\mathrm{Recall}}\;$$
2. Balanced error rate (BER):
  (9) $$\mathrm{BER}=\frac{1}{2}\cdot (\frac{\mathrm{FP}}{\mathrm{FN}+\mathrm{TP}}+\frac{\mathrm{FN}}{\mathrm{FP}+\mathrm{TN}})\;$$
3. Quality index (QI, Geometric mean):
  (10) $$\mathrm{QI}=\sqrt{\mathrm{Se}\cdot \mathrm{Sp}}\;$$
4. Matthews correlation coefficient (MCC):
  (11) $$\mathrm{MCC}=\frac{TP\cdot TN-FP\cdot FN}{\sqrt{\left(TP+FP)(TP+FN)(TN+FP)(TN+FN\right)}}\;$$

## 3. Results

**Figure 4:**
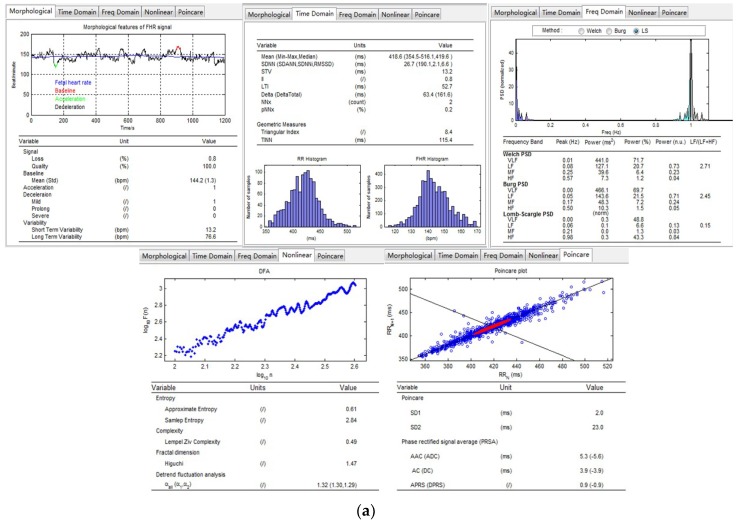

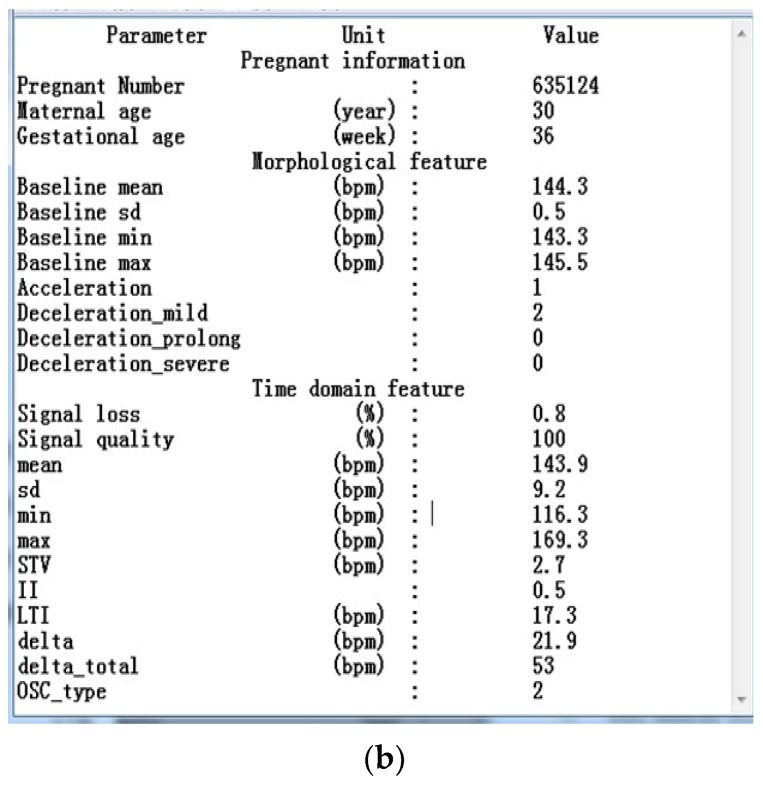
The results of the feature extraction algorithm of No. 1001 FHR recording (internel database number) using CAS-FHR. (**a**) The results are viewed in the software interface, inlcuding the morphological, time and frequency domain and nonlinear features; (**b**) The results are saved in txt format for further analysis.

The obstetricians were able to utilize the computerized software to analyze the FHR signals and obtain all the mentioned features automatically. Figure 4 shows a comprehensive list of the extracted features of the different domains in the form of tables and graphs.

For the CTU-UHB database, Table 3 and Table 4 present the distribution of four feature subsets for two classes before normalization. We considered *p* < 0.05 to indicate significance between each feature and fetal states, and the results showed that the features of meanBL, minBL, and maxBL of Set_1; meanRR, minRR, maxRR, and STV of Set_2; and FD_Hig, LZC, and DFA_alpha of Set_4 had statistical significance in identifying different fetal conditions with a 95% CI. Therefore, the ST reduced the original 47 features to the above 9 features, which were combined as Set_A.

**Figure 5:**
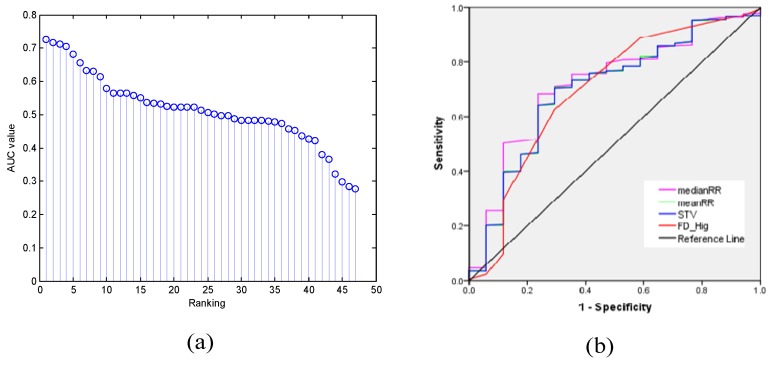
The result of feature selection using area under the curve (AUC). (**a**) The AUC value of a total of 47 individual features for classifying the fetal state in descending order; (**b**) The receiver operating characteristic (ROC) curve for the top four features used in the diagnosis of a pathological situation: MedianRR, AUC = 0.73 (95% CI, 0.62–0.86), *p* = 0.001; meanRR, AUC = 0.72 (95% CI, 0.60–0.84), *p* = 0.003; STV, AUC = 0.71 (95% CI, 0.60–0.83), *p* = 0.003; FD_Hig, AUC = 0.71 (95% CI, 0.59–0.83), *p* = 0.005.

**Figure 6:**
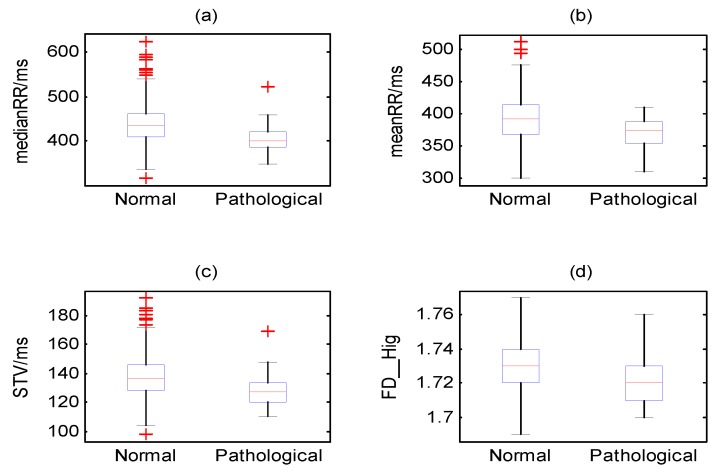
The distribution of normal and pathological classes for the four most highly ranked features (medianRR, meanRR, STV, and FD_Hig) using box plots.

In clinical practice, we wanted to know which features could better predict the fetal state as the independent variable. Consequently, it was necessary to rank the importance of features based on visual inspection. This study performed the ranking stage based on the value of the AUC, and the result is displayed in descending order, as shown in Figure 5a. More specifically, Figure 5b represents the ROC curve of the four 'best' features with an AUC value greater than 0.71 (medianRR, STV, meanRR, and FD_Hig). Therefore, this method reduced the original 47 features to the above 4 features, which were combined as Set_B. Furthermore, Figure 6 shows the distribution of the four non-normalized features for the two categories, and the differences in the median between two fetal classes were significant (*p* < 0.05, Wilcoxon rank sum test). 

**Figure 7:**
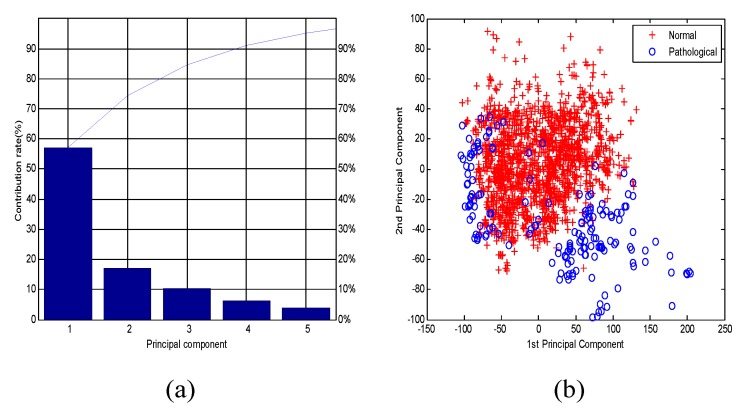
(**a**) Principal components (PCs) based on the contribution rate using principal component analysis (PCA); (**b**) The distribution between the first PC and second PC for two groups (normal and pathological).

**Figure 8:**
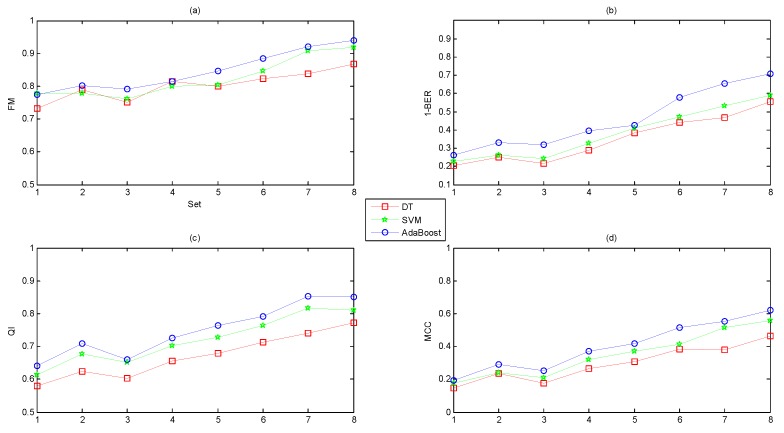
Comparison of the averaged performance evaluation indicators using different feature subsets based on three classifiers across ten folds. The x-axis shows Set_1, Set_2, Set_3, Set_4, Set_2 + Set_3, Set_1 + Set_2 + Set_3, Set_2 + Set_3 + Set_4, and Set_Complete.

As a typical dimensionality reduction method, PCA has demonstrated its power in pattern recognition before classification involving biological signals. Although PCA is a form of linear unsupervised technology, it continues to have a higher level of competitiveness when applied to real-life data for advanced schemes. Figure 7a shows that the first five principal components (PCs) were determined to have more than 95 percent contribution rates. Therefore, this method reduced the original 47 dimensions to 5 dimensons of the above PCs, which were combined as Set_C. And Figure 8b presents the distribution between the first PC and second PC for two fetal classes.

In summary, according to the components of Set_A, Set_B, and Set_C, we discovered that the ST, AUC, and PCA methods reduced the original 47 dimensions to 9, 4, and 5 dimensions, respectively, which reflected the effectiveness of dimensionality reduction.

Regarding the four measurements (FM, QI, MCC and BER), the first three showed a positive relationship with performance (higher values correspond to better classifiers); however, the higher the value of BER, the lower the performance was. Therefore, instead of the value of BER, we chose 1-BER as the measurement to transform this to a positive relationship to performance, as for the other three indicators. 

To prove the effectiveness of our proposed software, we compared the classification performance using different feature subsets based on various combinations of the four feature domains of the FHR signals. We concluded the following from Figure 8:

- Different feature domains contained different amounts of information regarding the fetal state.
- A combination of several feature domains could improve the performance, and the original feature set (Set_Complete) achieved the best performance.
- The classification capacity of AdaBoost was better than DT and SVM.

**Figure 9:**
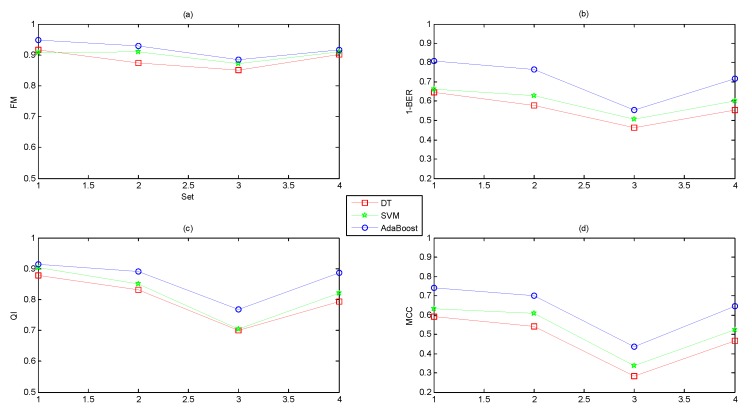
Comparison of the averaged performance evaluation indicators using different datasets obtained from ST, AUC and PCA based on three classifiers across ten folds. The x-axis shows Set_A, Set_B, Set_C, and Set_Complete.

Moreover, we also experimented with three datasets consisting of selected features, as illustrated in Figure 9. The following conclusions could be drawn:

- The feature selection algorithms of ST and AUC improved the performance while the dimensionality reduction method of PCA reduced the performance.
- The classification abilities of the three classifiers ranked in the following order: AdaBoost > SVM > DT.

**Figure 10:**
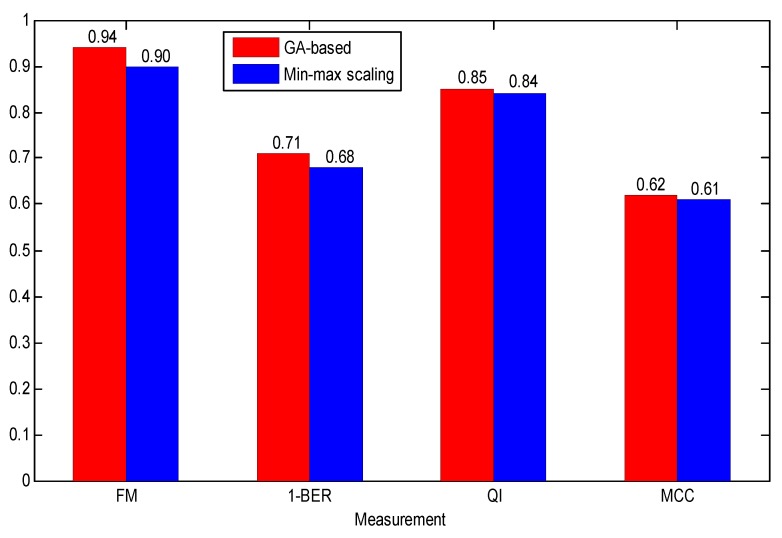
Comparison of the averaged performance evaluation indicators using different data normalization methods (GA-based and min-max scaling) based on the Set_Complete and AdaBoost.

Finally, based on the same dataset (Set Complete) and classifier (AdaBoost), we conducted the comparative experiment regarding the effect of different data normalization methods on classification performance. It can be observed from Figure 10 that the GA-based data normalization method achieved better performance than common min-max scaling, and demonstrated the relationship between the GA and FHR parameters.

## 4. Discussion

In this study, a comprehensive set of useful parameters originating from different domains (morphological, time and frequency, and nonlinear features) was extracted from the FHR signals using CAS-FHR. These features contained as much of the detailed physiological information as possible that could be associated with the fetal state. Several feature selection and ML algorithms were proposed to achieve the optimal classification performance in assessing fetal state.

The experimental results proved the effectiveness of our proposed approach, and several obvious conclusions were reached:

- Each feature domain reflected physiological information to different degrees: Nonlinear > time domain > frequency domain > morphological.
- The more features we used, the better the performance. A combination of 47 features yielded better performance than other combinations of individual feature subsets.
- The suitable feature selection algorithms (ST and AUC) improved the performance, but the dimensionality reduction approach (PCA) reduced the performance.
- The classification capacity of the ensemble learning algorithm (AdaBoost) was more powerful than common base classifiers (DT and SVM).
- Due to the relationship between the FHR parameters and GA, the GA-based data normalization method achieved better performance than common min-max scaling method.

In recent decades, many studies have attempted to predict fetal state accurately using the FHR signal, as shown in Table 5. It is difficult to compare these studies with each other due to the many factors involved, such as differences in the fetal diseases, databases and annotations. Fortunately, since the CTU-UHB became open access and available from PhysioNet several years ago, researchers have used this public database to test the performance of their respective methods. Table 5 shows that our proposed approach achieved better performance than the others by extracting comprehensive features and applying the ST and AdaBoost algorithms.

## 5. Conclusions

FHR recording allows obstetricians to monitor fetal status and adopt timely medical intervention before permanent damage is done to the newborn during pregnancy and delivery. Unfortunately, the visual interpretation of FHR traces using the naked eye may not be objective and reproducible for obstetricians. Therefore, computerized analysis represents a major advance in the early identification of prenatal pathologies. In addition, many ML and data mining techniques have been employed to classify FHR signals.

In this work, we proposed a novel software package to analyze FHR signals by means of extracting comprehensive features, including morphological, time and frequency domain and nonlinear features. Several feature selection (ST and AUC) and dimensionality reduction (PCA) methods were then designed to select the optimal features. The fetal state was classified by ML algorithms (DT, SVM, and AdaBoost). An open-access database was employed, and the umbilical artery pH was chosen as the objective criterion to classify the fetal state (normal or pathological). Compared to other approaches, our proposed approach (using ST and AdaBoost) yielded better performance in assessing fetal state with an Acc of 92%, a Se of 92%, a Sp of 90% and an FM of 95%. 

In summary, the highlights of the work are as follows: (i) The CAS-FHR software is proposed to analyze the FHR signal automatically and to extract features comprehensively; (ii) a suitable feature selection algorithm was proved to improve the performance; (iii) ensemble learning has a more powerful classification capacity; (iv) the GA-based data normalization method can improve performance; and (v) the result of this work can be considered a baseline for extracting more informative features and designing stronger classifiers.

As the paper title indicated, the primary innovative point of this work was proposing a comprehensive feature analysis method using FHR signal. We only selected three classifiers for the intelligent assessment of fetal state. Therefore, the research direction of future work is to explore the influence of different ML algorithms on the classification performance. In addition, we will integrate the ML algorithm in the CAS-FHR software to implement a medical decision support system, which can assist obstetricians in assessing the fetal state objectively and accurately in clinical practice.

## Figures and Tables

**Table 1:** An overview of the information available in the open access CTU-UHB cardiotocography (CTG) database.

Information	Mean	Min	Max
Maternal age (MA, year)	29.6	18	46
Gestational age (GA, week)	40.0	37	43
pH	7.23	6.85	7.47
BDecf (mmol/L)	4.60	−3.40	26.11
pCO_2_	7.07	0.70	12.30
BE	−6.38	−26.80	−0.20
Apgar 1 min	8.3	1	10
Apgar 5 min	9.1	4	10
Gravidity	1.4	1	11
Parity	0.4	0	7
Diabetes	No = 515, Yes = 37		
Birth weight (BW, g)	3401	1970	4750
Infant sex	Male = 286, Female = 266		
Delivery type	Vaginal = 506, Cesarean section = 46		

**Table 2:** Performance measurements for binary classification computed from a confusion matrix.

	Positive	Negative	Evaluation Focus
Predicted as positive	TP	FP	/
Predicted as negative	FN	TN	/
Acc	$\frac{\mathrm{TP}+\mathrm{TN}}{\mathrm{TP}+\mathrm{FP}+\mathrm{FN}+\mathrm{TN}}$		The overall efficiency of a classifier
Se (Recall)	$\frac{\mathrm{TP}}{\mathrm{TP}+\mathrm{FN}}$		The efficiency of a classifier to categorize positively labeled data
Sp	$\frac{\mathrm{TN}}{\mathrm{TN}+\mathrm{FP}}$		The efficiency of a classifier to categorize negatively labeled data
Precision	$\frac{\mathrm{TP}}{\mathrm{TP}+\mathrm{FP}}$		The data with positive labels correctly classified by the classifier

Note: Positive = normal, negative = pathological; TP = true positive, FP = false positive, FN = false negative, TN = true negative.

**Table 3:** Features extracted from the FHR signals for the normal and pathological groups (Set_1: Morphological; Set_2: Time domain). Key: Values are given as the mean (standard deviation). *p*-values were calculated using the Wilcoxon-Mann-Whitney nonparametric test with a significance level of 0.05.

Set	Parameter (Unit)	Normal (447)	Pathological (105)	*p*
Set_1	meanBL (bpm)	136.0 (14.4)	142.0 (16.2)	0.004
	sdBL (bpm)	2.4 (1.3)	3.2 (2.6)	0.318
	minBL (bpm)	132.0 (15.2)	135.9 (17.2)	0.008
	maxBL (bpm)	140.7 (14.7)	148.0 (16.6)	0.002
	ACC	1.45 (2.24)	2.24 (3.10)	0.185
	DEC_mild	1.40 (1.84)	2.0 (1.85)	0.098
	DEC_prolong	0.07 (0.30)	0.17 (0.47)	0.657
	DEC_severe	0.03 (0.19)	0.00 (0.00)	1.000
Set_2	meanRR (ms)	447.4 (43.3)	431.0 (52.2)	0.005
	minRR (ms)	370.4 (31.8)	349.9 (27.2)	0.019
	maxRR (ms)	640.5 (158.2)	654.1 (165.8)	0.341
	medianRR (ms)	439.3 (40.6)	418.5 (50.2)	0.002
	SDNN (ms)	42.6 (27.8)	51.4 (32.7)	0.896
	SDANN (ms)	29.7 (24.1)	38.3 (27.4)	0.983
	SDNNi (ms)	25.1 (14.5)	28.9 (18.1)	0.879
	RMSSD (ms)	10.7 (5.4)	12.0 (7.3)	0.912
	NNx	12.5 (16.0)	17.5 (22.2)	0.596
	pNNx	1.1 (1.3)	1.5 (1.9)	0.596
	STV (ms)	12.1 (8.7)	14.1 (17.6)	0.005
	II	0.9 (0.2)	0.9 (0.2)	0.079
	LTI (ms)	640.5 (158.2)	654.1 (165.8)	0.732
	delta (ms)	82.5 (42.3)	92.9 (55.2)	0.746
	delta_total (ms)	270.1 (154.6)	304.2 (158.3)	0.927
	FHRVTi	6.2 (2.6)	6.6 (2.8)	0.394
	TINN	81.7 (40.6)	81.9 (37.7)	0.978

**Table 4:** Features extracted from the FHR signals for the normal and pathological groups (Set_3: Frequency domain; Set_4: Nonlinear). Key: Values are given as the mean (standard deviation). *p*-values were calculated using the Wilcoxon-Mann-Whitney nonparametric test with a significance level of 0.05.

Set	Parameter (Unit)	Normal (447)	Pathological (105)	*p*
Set_3	Power_VLF (ms^2^)	1477 (2747)	2708 (4734)	0.757
	Power_LF (ms^2^)	639 (998)	927 (1464)	0.359
	Power_MF (ms^2^)	180 (233)	201 (300)	0.527
	Power_HF (ms^2^)	105 (139)	120 (173)	0.536
	Power_Total (ms^2^)	2401 (3707)	3956 (5989)	0.498
	Percent_VLF (%)	86.3 (8.4)	87.5 (8.3)	0.513
	Percent_LF (%)	11.1 (7.0)	10.1 (6.9)	0.256
	Percent_MF (%)	1.8 (1.2)	1.8 (1.2)	0.935
	Percent_HF (%)	0.8 (0.7)	0.7 (0.5)	0.382
	Ratio_Band	4.6 (1.7)	4.4 (1.5)	0.340
Set_4	FD_Hig	1.54 (0.09)	1.52 (0.11)	0.005
	ApEn	0.41 (0.00)	0.41 (0.00)	0.168
	SampEn	2.44 (0.38)	2.37 (0.35)	0.102
	LZC	1.13 (0.11)	1.14 (0.12)	0.046
	Hurst	0.93 (0.04)	0.94 (0.03)	0.099
	alpha	1.32 (0.12)	1.32 (0.13)	0.006
	ACC (ms)	8.95 (8.97)	8.54 (8.74)	0.603
	ADC (ms)	−8.10 (8.88)	−8.35 (8.21)	0.449
	APRS	2.98 (2.24)	2.78 (2.56)	0.774
	DPRS	−2.77 (2.29)	−2.45 (2.73)	0.856
	SD1 (ms)	8.45 (5.56)	8.76 (5.21)	0.579
	SD2 (ms)	54.57 (44.59)	55.13 (46.32)	0.548

**Table 5:** Summary of recent work on the discrimination of fetal pathological conditions using FHR signals. (N = normal, P = pathological, FE = feature extraction, FE: Feture selection, C: Classifier).

Reference (Year)	Database	Distribution (N/P)	Method	Performance (%)
[7] (2011)	Private	Imbalance (30/60)	FE: Empirical mode decomposition C: Support vector machine	Acc = 87
[8] (2012)	Private	Imbalance (123/94)	FE: 12 (nonlinear) FS: Information gain C: Support vector machine, naïve Bayes, C4.5	Se = 73 Sp = 76 FM = 71
[54] (2014)	Private	Balance (255/255)	FE: 64 (morphological and linear) FS: genetic algorithm C: Support vector machine	Se = 83 Sp = 66
[9] (2015)	Private	Imbalance (30/15)	FE: Ratio and Hurst C: Statistical test (*p*-value)	AUC = 81
[55] (2014)	CTU-UHB	Imbalance (175/377)	FE: 33 (morpholical, linear and nonlinear) C: Latent class analysis+random forest	Se = 72 Sp = 78
[56] (2017)	CTU-UHB	Imbalance (508/44)	FE: 42 (morpholical, linear and nonlinear) FE: Relevance in estimating features C: Least square support vector machine	Se = 72 Sp = 65
[10] (2018)	CTU-UHB	Imbalance (439/113)	FE: Image-based time-frequency features FS: genetic algorithm C: Least square support vector machine	Se = 63 Sp = 66
Current work	CTU-UHB	Imbalance (447/105)	FE: 47 (morpholical, linear and nonlinear) FS: Statistcal test, AUC C: Adaptive boosting	Acc = 92 Se = 92 Sp = 90 AUC = 91
